# Death at Watering Places

**Published:** 1874-07

**Authors:** 


### DEATH AT WATERING PLACES.

Long Branch and the camp meeting grounds of Martha's Vineyard have the houses built on an almost dead level. Within a few rods of one of the Long Branch hotels in the rear are about fifty privies; at Martha's Vineyard the cottages are scattered around in every direction, with a thin undergrowth of small trees and bushes; in these "clumps" are found the privies of each house; some of them have none, and the occupants deposit bodily offal wherever the leaves are thick enough to obstruct the view of passers by. In the course of time the soil must be saturated, and any water drawn from wells must necessarily contain, by the laws of drainage and of specific gravity, as applied to water thrown on the ground, more or less of the solid matters of the privy and the water closet. To be on the safe side the proprietors of the hotels at both places should use only cistern water for drinking and cooking; and, in addition, should spend a hundred thousand dollars, if necessary, to have a plentiful supply of underground drains, to carry off all the surface water of these places direct into the sea, with as great a declination as possible, in order to promptly convey all matters into the ocean. Every rain softens and dissolves into a more or less fluid form all that is deposited on the surface of the earth; hence, the idea of drinking any water at these places from a well or spring, or even an underground cistern, is most revolting; and the great pleasure seeking public have a right to demand attention to these matters. Every person of intelligence ought to take it upon himself to have ocular demonstration that the water supplied at the hotels is from unobjectional sources.

Medical journals at home and abroad abound in details of the havoc which diseases of a typhoid character have made in whole families—the cause of which, on scientific investigation, has been demonstrated beyond a doubt to have been laid in a defective privy drain passing near a well or spring of running water.

People living in flat localities, in neighborhoods, are dying every year—more in the old world than in the new—because older and more thickly inhabited, in consequence of a want of drainage in this connection, Persons who have travelled abroad well know that it is almost impossible to get any water fit to drink; this may be a fact rather than a prejudice, from the two circumstances that so many millions of people have been buried in the ground in the circuit of the ages, and the offal of other millions is deposited daily on the surface of the earth ; and as a privy may drain into a well half a mile off, to say nothing of stables, and barns, and cattle pens, the safer plan is to drink water from above ground—cisterns filled from the roofs of houses.

